# SASH1 promotes melanin synthesis and migration via suppression of TGF-β1 secretion in melanocytes resulting in pathologic hyperpigmentation

**DOI:** 10.7150/ijbs.38415

**Published:** 2020-02-10

**Authors:** Hongzhou Cui, Shuping Guo, Hongxia He, Huina Guo, Yuliang Zhang, Binquan Wang

**Affiliations:** 1 Department of Dermatology, the First Hospital, Shanxi Medical University, Taiyuan, Shanxi, China; 2 Shanxi Key Laboratory of Otorhinolaryngology Head and Neck Cancer, Shanxi Medical University, Taiyuan, Shanxi, China; 3 The Key Scientific and Technological Innovation Platform for Precision Diagnosis and Treatment of Head and Neck Cancer, Shanxi Province, Taiyuan 030001, Shanxi, China

## Abstract

Dyschromatosis universalis hereditaria (DUH) is an autosomal dominant pigmentary genodermatosis characterized by the presence of patches of hyperpigmentation and hypopigmented macules distributed over the body, with most cases reported in Asia. DUH is a heterogeneous disease and a small portion of patients carry the ABCB6 variant. In the present study, exome sequencing of four generations of a Chinese family with DUH identified a c.1761C>G (p.Ser587Arg) mutation in exon 15 of SAM and SH3 domain containing 1 (SASH1) that was found to co-segregate in some family members. Immunohistological analysis of biopsy specimens showed that SASH1 was diffusely distributed in all layers of the epidermis, suggesting increased transepithelial migration of melanocytes (MCs). The point mutation c.1761C>G of SASH1 was successfully induced in immortalized human melanocyte (PIG1) cells, which resulted in the downregulation of SASH1 expression. Bioinformatics analysis showed that mutated SASH1 downregulated thrombospondin 1 (THBS1) expression and inactivated transforming growth factor beta 1 (TGF-β1) signaling. TGF-β1 expression by PIG1cells was found to negatively regulate SASH1 protein expression. Transwell migration and wound-healing assays showed an increase in the migration and invasion capabilities of the cells carrying the mutation. Further, SASH1 mutations induced downregulation of melanin content. The study results suggest cross-talking between SASH1-TGF-β1 signaling, demonstrating the proposed MC migration modulation models and affecting melanin trafficking in the epithelium.

## Introduction

Dyschromatosis universalis hereditaria (DUH, OMIM #127500) is a rare genodermatosis that manifests as irregularly shaped, asymptomatic hyper- and hypopigmented macules that appear in infancy or early childhood and are distributed on the trunk, limbs, and sometimes the face. Ichigawa first described DUH in a Japanese family in 1933 1. Since then, more than 100 cases of DUH have been reported worldwide, with more than three-quarters occurring in Asia 2.

DUH is an autosomal-dominant inheritable disease that has been linked to three chromosome segments: 6q24.2-q25.2 in two Chinese families 3-4, 12q21-q23 in an Arab family 5, and 2q33.3-q36.1 in a Chinese family. Subsequently, the ATP binding cassette subfamily B member 6 (ABCB6) gene located at 2q33.3-q36.1 was found to be the cause of DUH 6.

A recent study by our group found that none of the members of four generations of a Chinese family with DUH carried the ABCB6 mutation. Exome sequencing identified the SAM (sterile alpha motif) and SH3 (Src homology domain 3) domain containing 1 (SASH1) genes as the cause of DUH. SASH1 has been identified as a candidate tumor suppressor gene in several cancers 7-9. Reduced expression of SASH1 is associated with aggressive tumor growth, metastasis formation, and poor patient survival. Increasing evidence has shown that different signaling pathways, including PI3K/Akt and TGF-β1, induce morphological cellular changes and alterations in cell migration and invasion consistent with the differential expression of SASH1 10,11. Interestingly, several studies have reported that SASH1 plays an important role in promoting tumor cell invasion 10,12. Thus, SASH1 may have various specific functions in different cell types. SASH1 has been investigated in cases of dyschromatosis symmetrica hereditaria, a phenotype that overlaps with the pattern of skin anomalies in DUH. Reduced E-cadherin expression in SASH1 variants has been interpreted as a novel mechanism that modulates the migration and localization of MCs 13. In addition, previous studies reported that SASH1 variants were associated with different pigmentation disorders 14-17. These investigations determined that SASH1 was involved in the regulation of human skin pigmentation. However, the biological role of SASH1 in DUH remains largely unknown.

Here, we identified a SASH1 variant associated with the DUH phenotype in a Chinese family and further explored the possible mechanism of SASH1-mediated signaling/pathway changes in melanin synthesis and metabolism. A synthetic model used for prediction of mRNA expression profiles showed that SASH1/TGF-β1signaling was involved in cell migration and that THBS1 probably participates in this cascade to modulate melanin production and melanocyte (MC) transport.

## Materials and Methods

### Subjects

**Figure 1:**
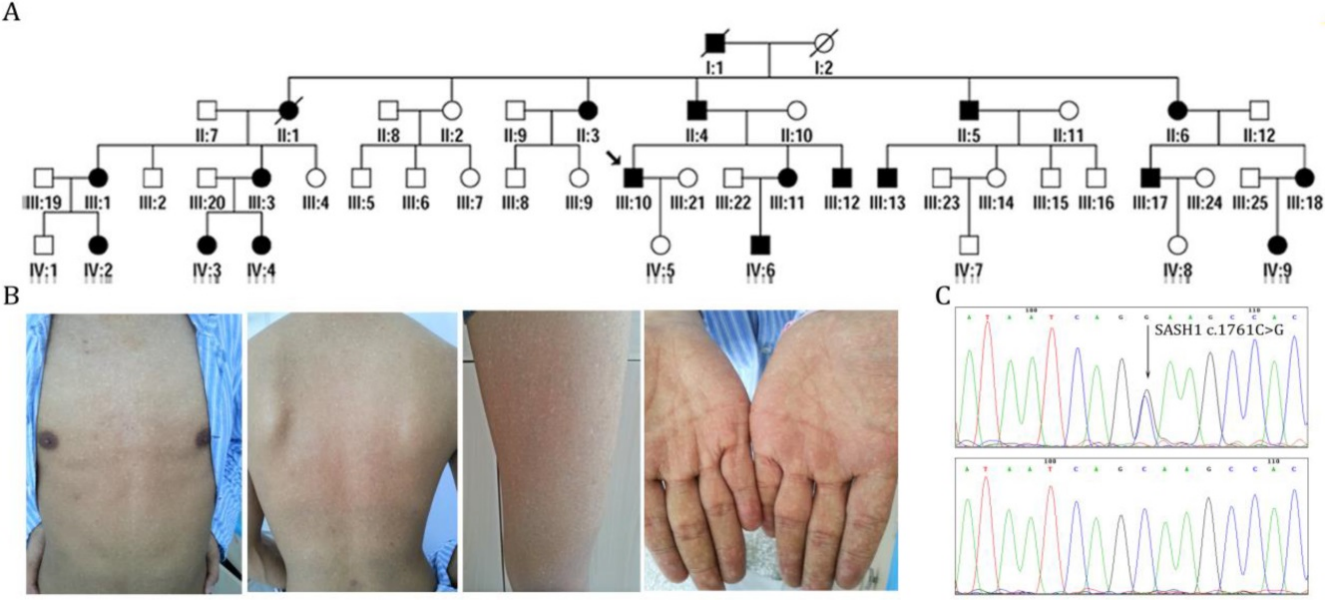
** Identification of SASH1 as the causative mutation in DUH in a Chinese family. (A)** Pedigrees of members of a Chinese family with DUH. **(B)** Clinical features of the proband. **(C)** Mutation analysis identified c.1761C>G (p.Ser587Arg) in SASH1 in DUH.

We characterized the members of four generations of a Chinese family with DUH, in which the disease was transmitted in an autosomal dominant manner (Figure 1A). The age at onset in this family ranged from 12 to 41 years. The proband (III:10) was a 39-year-old man who had normal skin at birth. Hyperpigmented and hypopigmented macules appeared initially on his forehead at the age of 12 years, and then gradually extended to involve his face, neck, forearms and dorsal hands, trunk, and legs. The lesions stopped advancing at the age of 19 years. He experienced no pruritus or pain. Examination of the skin showed motley hyperpigmented and hypopigmented macules that nearly involved his whole body. The lesions occurred in symmetrical patterns and were most obvious on the face, neck, trunk, forearms, and the dorsa of his hands (Figure 1B). His palms, soles, oral mucosa, hair, nails, and teeth were normal. Histopathological examination of a skin biopsy from his back revealed the presence of dense pigment granules in the basal layer of the epidermis, with no obvious increase in MCs, although melanophages were observed in the upper dermis. In the hypopigmented area, there were few or no pigment granules with no obvious reduction in the abundance of MCs. None of the affected members in this family had skin cancer or any other systematic disease. Skin and blood tissues were processed after obtaining informed consent. The study protocol was approved by the Ethics Committee of the First Hospital of Shanxi Medical University and was conducted in accordance with the tenets of the Declaration of Helsinki.

### Exome capture and sequencing

The exomes of the proband (III:10) and one unaffected individual (II:2) were sequenced using the Agilent SureSelect Human All Exon V6 Kit (Agilent Technologies, Santa Clara, CA, USA). Briefly, purified genomic DNA samples were randomly fragmented by Covaris, Inc. (Woburn, MA, USA). Ligation-mediated polymerase chain reaction (PCR) was used to amplify the extracted DNA after purifying with Agencourt AMPure XP beads (Beckman Coulter, Fullerton, CA, USA). The PCR products were added to the SureSelect Biotinylated RNA Library to acquire enriched hybridized fragments. Each captured library was then loaded on a HiSeq PE150 platform (Illumina, Inc., San Diego, CA, USA). Read pairs were aligned to a reference human genome (GRCh37/hg19) using Burrows-Wheeler Aligner version 0.7.8-r455 (http://bio-bwa.sourceforge.net/).

### Variant calling and annotating

Single nucleotide variants (SNVs) and indels were called for all exomes jointly using the GATK HaplotypeCaller. Called variants with a quality score of >30 were accepted. A truth sensitivity cutoff of 99.0% was used for both SNVs and indels. Additional filtering was applied to exclude variant sites where >80% of the samples had a read depth of <5. Coding variants were annotated and categorized as non-synonymous, splice acceptor and donor site mutations, or coding indels. Considering the dominant inheritance patterns of the underlying mutations in the study subjects, compound heterozygous variants with allele frequencies of <1%were included in the dbSNP Build 147 and 1000 Genomes Project, NHLBI-ESP project, 60706 Exome Aggregation Consortium and in-house genome and exome analyses. To exclude likely benign amino acid changes, missense variants were considered only if two or more of the four following *in silico* methods predicted the variant to be deleterious: CADD64, PolyPhen-2 HumVar65, SIFT66, and MutationTaster. The variants located in the reported linkage intervals were first chosen and the candidate variants were verified in other pedigree members.

### Cell culture

Immortalized human melanocyte (PIG1) cells were purchased from ATCC (Manassas, VA, USA) and authenticated by short tandem repeat profiling. Human embryonic kidney 293T cells were obtained from Shanghai GeneChem Co., Ltd. (Shanghai, China). All cell lines were maintained in Dulbecco's Modified Eagle's Medium (HyClone Laboratories, Inc., Logan, UT, USA) supplemented with 10% fetal bovine serum (HyClone Laboratories, Inc.,) and cultured at 37ºC under a humidified atmosphere of 95% air/5% CO_2_. Primary antibodies against SASH1 (bs-6099R; Bioss Antibodies, Beijing, China), TGF beta 1 (ab92486; Abcam, Shanghai, China), FN1 (ab6328, Abcam, Shanghai, China), and glyceraldehyde 3-phosphate dehydrogenase (GAPDH; Santa Cruz Biotechnology, Inc., Dallas, TX, USA), as a loading control, and ahorseradish peroxidase-conjugated secondary antibody against immunoglobulin G (Santa Cruz Biotechnology, Inc.) were used for immunoblotting.

### Sanger sequencing and mutation analysis

The primers used for validation of the SASH1 variants and mutation analysis are listed online in Supplementary Table 4. PCR amplification was carried out using an ABI 9700 Thermal Cycler (Applied Biosystems, Carlsbad, CA, USA). The PCR products were purified using a QIAquick PCR Purification Kit (Qiagen, Hilden, Germany) and sequenced on an ABI PRISM 3730 automated sequencer (Applied Biosystems). Chromas 3.0 version was used to read the amplification fragments.

### Immunohistochemical (IHC) analysis

Epithelial tissues from affected individuals with the S587R SASH1 mutation from pedigree family I were fixed in 10% formalin at 4°C for 24 h and then embedded in paraffin. The paraffin-embedded sections (5 μm) were incubated at 56°C overnight and then deparaffinized and rehydrated using xylene and an ethanol gradient. The sections were incubated with rabbit polyclonal antibody against SASH1 (bs-6099R; Bioss Antibodies). Finally, the sections were counterstained with hematoxylin and imaged under a microscope.

### Construction of SASH1 vectors

The SASH1 and its point mutation construct were cloned into the pcDNA3 basic vector. The point mutation construct pcDNA3-SASH1 C1761G, originating from the construct pcDNA3-SASH1, was amplified by PCR. The point mutation construct was created using a Site Directed Mutagenesis Kit (Beyotime Institute of Biotechnology, Haimen, China). The following primer sequences were used for PCR amplification of SASH1 C1761G: (forward) 5'-atcatcgatataatcagGaagccacccatgggg-3' and (reverse) 5'-ccccatgggtggcttcctgattatatcgatgat-3'. The plasmid containing SASH1 c.1519T>G (p.Ser507Ala) mutant CDS was synthesized by Synbio Technologies Company (Suzhou, China). All constructs were confirmed by sequencing (Figure 3A).

The pLenti6/SASH1-wild type (WT-SASH1) and pLenti6/SASH1-S587R (S587R-SASH1) vectors were constructed with primer pairs containing the *Age*I/*Eco*RI restriction enzyme sites. The constructed recombinant plasmids were identified by endonuclease digestion and sequence analysis. 293T cells were transfected in 10-cm plates with 20 μg of the expression recombinant plasmid, 25 μg of the pHelper, and 9 mg of self-compound transfection mix (Shanghai GeneChem Co., Ltd.). The transfected cells were cultured for 48-72 h to allow for viral secretion. Supernatants were then filtered using 0.45-mm filters. The lentivirus quality was tested by immunofluorescence analysis. The moderate virus titer fluorescence rate reached 80% for the infection of target P1G1 cells. Transduction was conducted using polybrene and enhanced infection solution (Shanghai GeneChem Co., Ltd.). After culturing for 72 h, the cells with good morphology were collected and once fluorescence rate reached 80% for RNA extraction and reverse transcription, quantitative PCR (qPCR) was performed to determine the knockdown efficiency.

### Microarray analysis of mRNA expression

Total RNA from WT-/S507A-/S587R-SASH1 PIG1 cells culture lysates was isolated with TRIzol reagent in accordance with the manufacturer's protocol (Invitrogen Corporation, Carlsbad, CA, USA). Transcription and cDNA target preparation were conducted using the IVT expression kit (Affymetrix, Santa Clara, CA, USA). Total RNA not exceeding 3 µg was further fragmented and labeled using the GeneChip WT Terminal Labeling and Controls Kit (Affymetrix). The labeled cDNA was hybridized onto GeneChip PrimeView Human Gene Expression Arrays using the GeneChip Hybridization, Wash, and Stain Kit according to the manufacturer's instructions (Affymetrix). After washing and staining with the Affymetrix GeneChip Fluidics Station 450 system, the arrays were scanned with an Affymetrix GeneChip Scanner 3000. The qualified data were normalized with the robust multi-array average algorithm and then log-transformed followed by median-subtraction. Significantly differentially expressed miRNAs between two groups were identified based on a false discovery rate threshold of less than 0.05 and a 2.0-fold change between the two groups. Functional pathway analysis was conducted using the commercially available software Ingenuity Pathways Analysis, according to the manufacturer's instructions. Upstream regulators, diseases and functions, regulators effects, and molecules in network analyses were predicted using commercially available software (Ingenuity Pathways Analysis), according to the manufacturer's instructions.

### Real-time qPCR

Total RNA was isolated using the same protocol as above. Equal amounts of RNA were reverse-transcribed into cDNA using the ReverTra Ace-a-First strand cDNA synthesis kit (M-MLV Reverse Transcriptase, Promega Corporation, Madison, WI, USA), as instructed by the manufacturer. Quantitative real-time PCR was performed using the Roche LightCycler 480 II System (DRR041A; Takara Bio, Inc., Otsu, Shiga, Japan) with SYBR Premix Ex Taq^TM^ reagent (DRR041A; Takara Bio, Inc.). The quantity of each mRNA was normalized to that of GAPDH mRNA, and relative abundance was calculated using the 2^-ΔΔCT^ method.

### Transwell migration and wound-healing assays

A transwell migration system with polycarbonate filters with 8.0-μm pores and an 8-μm Matrigel® Invasion Chamber (BD Biosciences, San Jose, CA, USA) was used to access the migration of MCs transfected with the mutant and WT SASH1 vectors. Briefly, 1×10^5^ cells were placed in the upper compartment, while DMEM (0.6 ml) containing 10% FBS was added to the lower compartment. The migrated cells were stained with Giemsa dye and photographed under a bright-field microscope after incubation for 24 h. For the wound-healing assays, 5×10^5^ cells were cultured on a cell plate for 24 h and then subjected to serum starvation. Then, 3-5 parallel lines were drawn on the plate using a 200-mL pipette. After washing three times with phosphate-buffered saline, the scratched cells were removed. After 24 h of culture, the plates were photographed to assess cell migration by quantitation of the distance that the cells had migrated within the lines. All experiments were repeated three times.

### Detection of melanin content

The pigmentation shift of PIG1 cells in the exponential stage was assessed after 56 h in the presence of 5% fetal calf serum. Cells were treated with 0.25% pancreatin and melanin particles were dissolved in 200 μL of 1 mol/L NaOH with the addition of 10% dimethyl sulfoxide. The absorbance value was measured using a microplate reader at a wavelength of 490 nm and the relative melanin content was determined by calculating the mean ratio of the PIG1 cells to that of the blank group.

### Statistical analysis

The WT-/S507A-/S587R-SASH1 vector and transfect experiments were totally repeated for 3 times at different time. Triplication for the microarray analysis in each group. WB assay, qPCR, transwell migration, wound-healing and melanin content assays were repeated 3 times independently, with 3 repetitions for each time. Data are expressed as the mean ± standard deviation. Statistically significant differences between two groups were identified using the Student's *t*-test, while comparisons among more than two groups were performed using one-way analysis of variance. A probability (*p*) value of < 0.05 was considered statistically significant.

## Results

### Identification of SASH1 as the causative gene of DUH

Sanger sequencing revealed that none of the patients carried ABCB6-coding variants. After exome capturing, an average of 14.22 Gb of paired-end, 150-bp length reads per individual were obtained and 99.0% of the target covered at least 10-fold (Supplementary Table 1). By combinatorial filtering with databases supplemented by bioinformatics analysis, 463 rare frequency variants (324 SNVs and 139 indels) were identified that had predicted deleterious variants with an autosomal-dominant genetic pattern. Subsequently, we screened five heterozygous mutations in five genes (*CPS1, SASH1, PTPRQ, CEP290 and C12orf42*) that were located in the loci reported in previous linkage studies. Among these variants, one missense variant (c.1761C>G, p.Ser587Arg) of SASH1 had co-segregated with the phenotype in the family (Figure 1C). This variant lies in exon 15 of SASH1 and replaces a highly conserved serine residue with an arginine residue at position 587. Further direct DNA sequence analysis of 300 control individuals or the public SNP databases on the HapMaps and UCSC Genome Browser websites, suggest that SASH1 is the causative gene of DUH (Supplementary Table 2-3).

### IHC analysis revealed that the SASH1 proteins were localized in multiple epithelial layers of the affected epidermis

**Figure 2:**
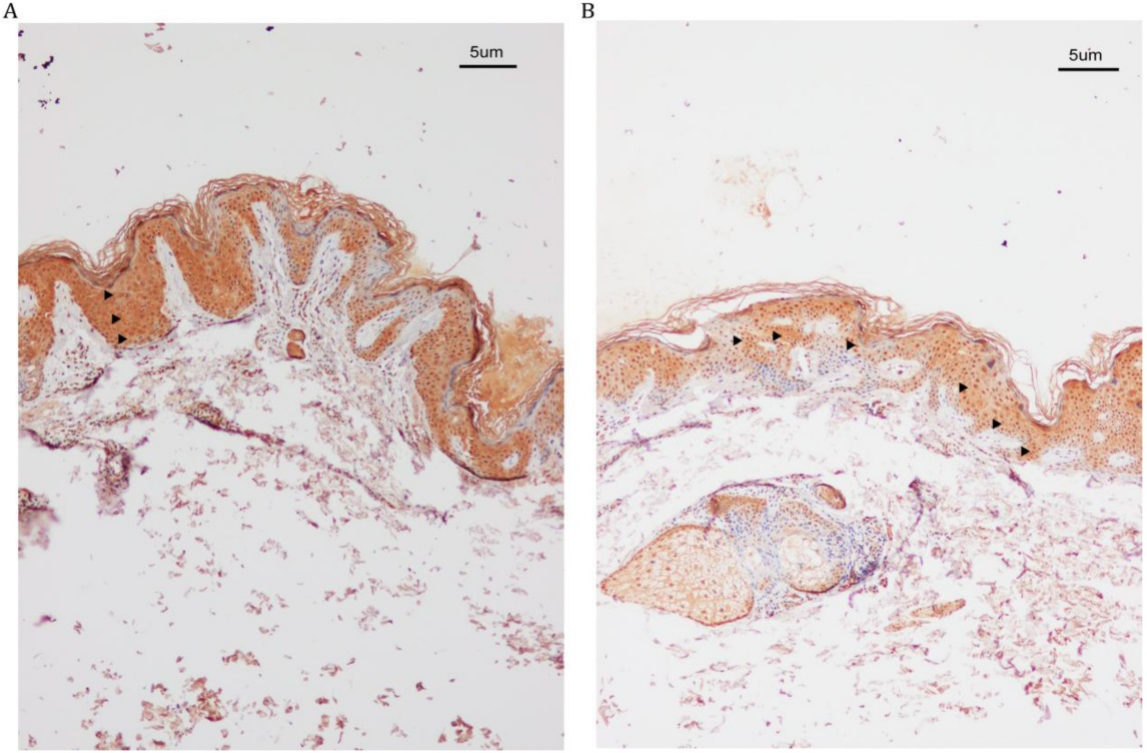
** IHC analysis of SASH1 expression in the DUH affected and unaffected individuals. (A)** SASH1 protein expression in multiple epithelial layers of a DUH lesion. **(B)** The normal and homogeneous levels of SASH1 were mainly distributed in the basal and nearby layers of the epidermis of unaffected individuals.

IHC analysis revealed increased SASH1 expression in DUH-affected epithelial tissues as compared to the unaffected epidermis. We also observed a heterogeneous distribution of MCs in some regions of the affected epidermis, while SASH1-positive cells were wildly distributed in multiple epithelial layers of DUH-affected individuals. However, in the unaffected epidermis, SASH1 expression was mainly concentrated in the under layer of the epidermis (Figure 2).

### SASH1 S587R mutation downregulate SASH1 expression

**Figure 3:**
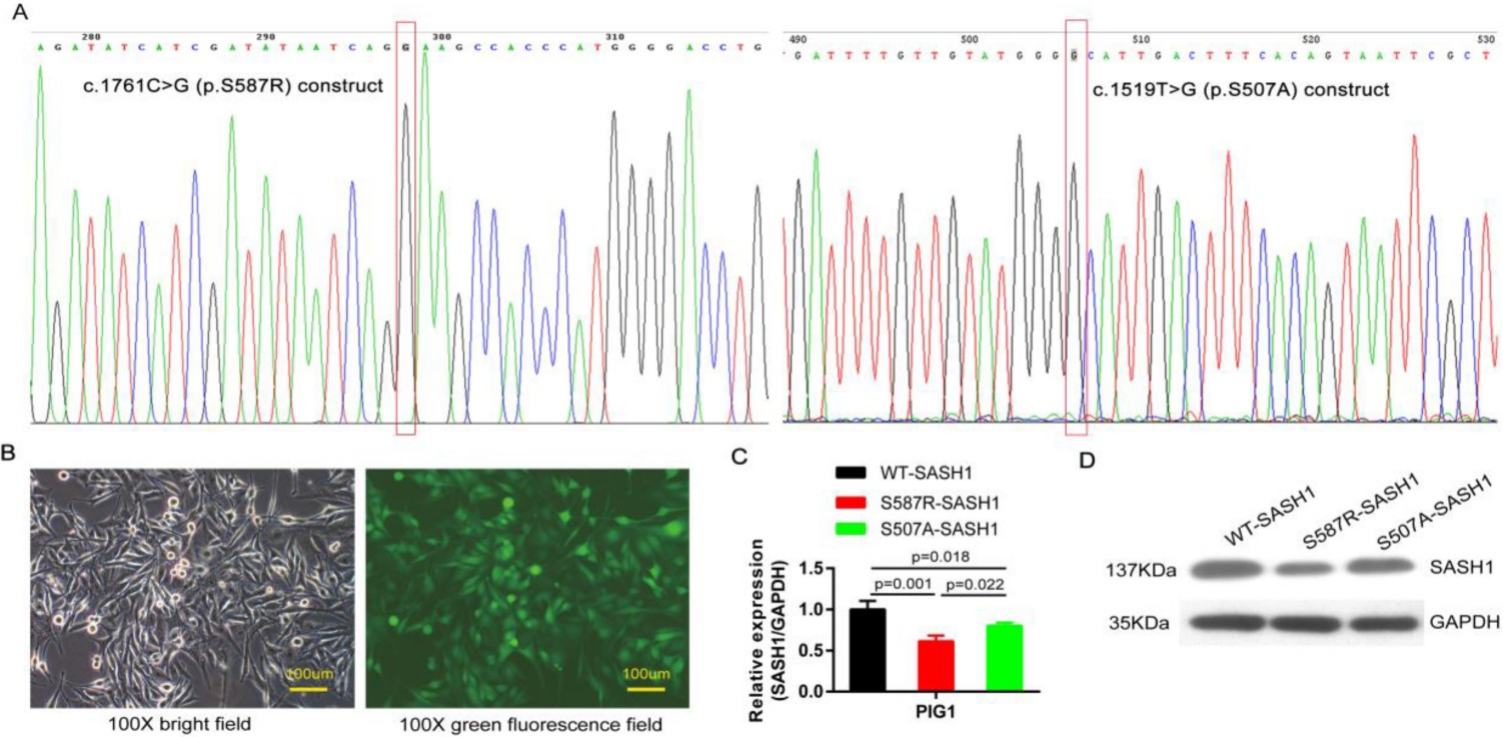
** SASH1 mutation down-regulated SASH1 expression in PIG1 cells. (A)** The c.1761C>G point mutation of the construct pcDNA3-SASH1 and SASH1 c.1519T>G mutant was confirmed by sequencing. **(B)** Bright field and green fluorescence field showed the point mutant in PIG1 cells after incubation for 72 h with a lentiviral RNAi vector. **(C)** SASH1 mRNA expression was down-regulated by 38.7% and 28.6% in S587R-SASH1 PIG1 cells, as compared to WT-SASH1 and S507A-SASH1 PIG1 cells. **(D)** Western blot analysis indicated that SASH1 protein expression of S587R-SASH1 PIG1 cell was down-regulated by 50.6% and 38.1%, compared with WT-SASH1 and S507A-SASH1 cells.

PIG1 cells were readily infected with lentiviruses containing blank and the mutant S507A-, S587R-SASH1, demonstrating that SASH1 can be stably expressed in PIG1 cells (Figure 3B). The PIG1 cells transfected with the mutant S587R-SASH1 exhibited significantly reduced SASH1 mRNA expression by 38.7% and 23.6%, as compared with cells expressing WT-SASH1 and S507A-SASH1 (*p*<0.05). Expression of the SASH1 protein was also reduced by 50.6% and 38.1%, respectively (Figure 3C-D).

### Gene expression profile analysis revealed that THBS1 is correlated with the mediation of cell migration through the SASH1-TGF-β1 pathway

**Figure 4:**
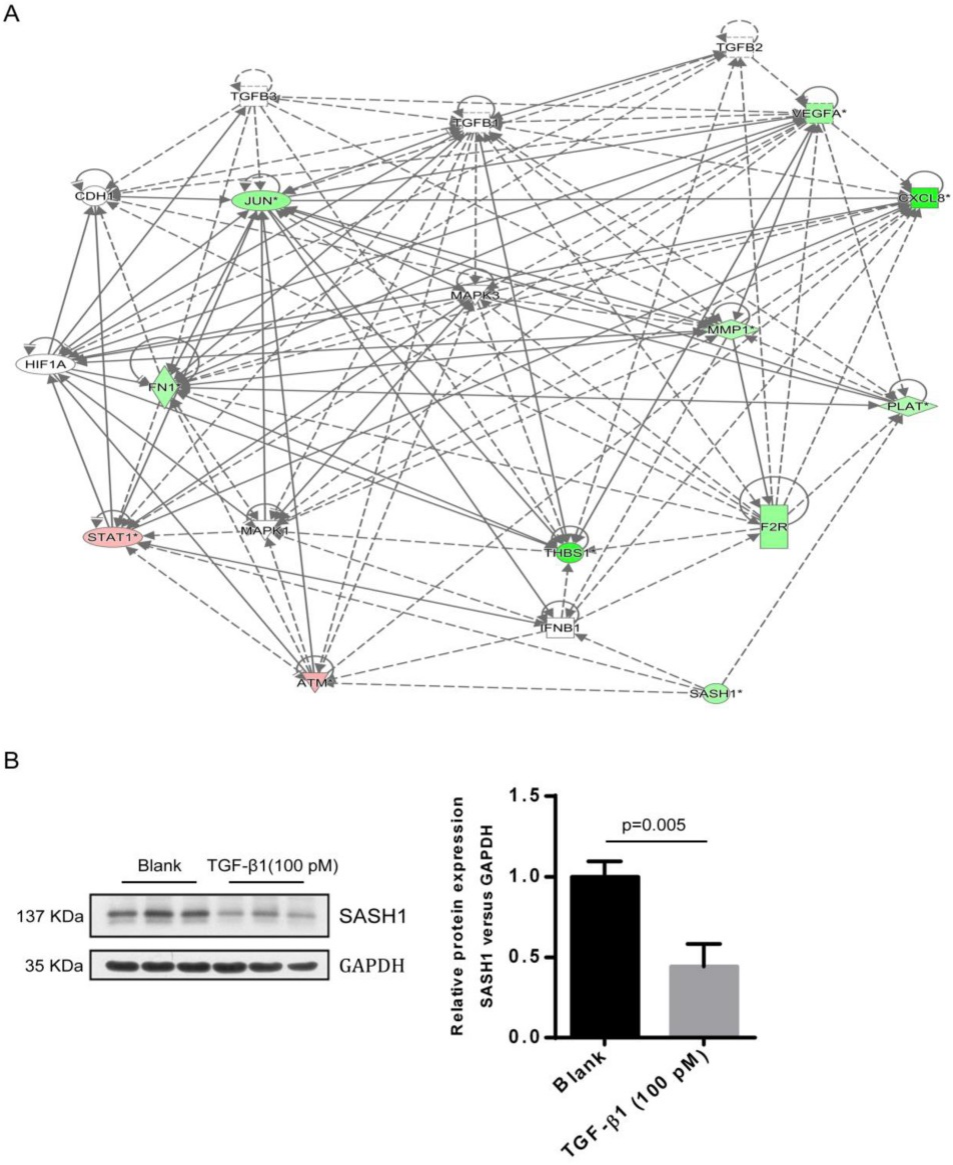
** A interaction between SASH1 and TGF-β1 signaling were identified by bio-informatics analysis and western blot. (A)** Interaction analysis illustrated the interrelationships among the molecules mainly affects cell migration. The data presented in red/green represent the up-/down-regulated expression, solid lines and arrow represent direct activation. **(B)** Western blot analysis of SASH1 protein expression in PIG1 cells treated with TGF-β1.

As compared to the WT-SASH1 PIG1 cell group, a total of 211 up-regulated genes and 304 down-regulated genes were identified in S587R-SASH1 PIG1 cells. Among the differentially expressed genes, 28 functional classifications, as annotated by gene ontology, were significantly enriched, which included transcription, nucleotide binding, and metabolic process, among others. Pathway analysis showed that the differentially expressed genes were significantly enriched in eight pathways (|Z-score|>2). Analysis of the upstream regulators of the differentially expressed genes showed that TGF-β1 was downregulated by 5.065-folds after SASH1 mutation and 54 genes were downregulated that were correlated consistently to SASH1 levels (Supplementary Figure 1). Notably, the TGF-β1 pathway was one of the top modulated canonical pathways following SASH1 mutation involved in cell migration (Z-score=-2.121). Next, we decided to focus on the TGF-β1 signaling involved in cell migration. Regulator effect network analysis revealed reliable relationships between the differentially expressed genes involving TGF-β1 and the possible upstream regulators, which predicted the inhibition of cell migration (Supplementary Figure 2). The interaction network analyses of the differentially expressed genes affecting cell migration suggested that the effects of TGF-β1 on mutant S587R-SASH1 PIG1 cells may be mediated through THBS1 overexpression and that THBS1 may play a critical role in the migration of MCs (Figure 4A).

### SASH1 mutation increases cell mobility and upregulates melanin content

**Figure 5:**
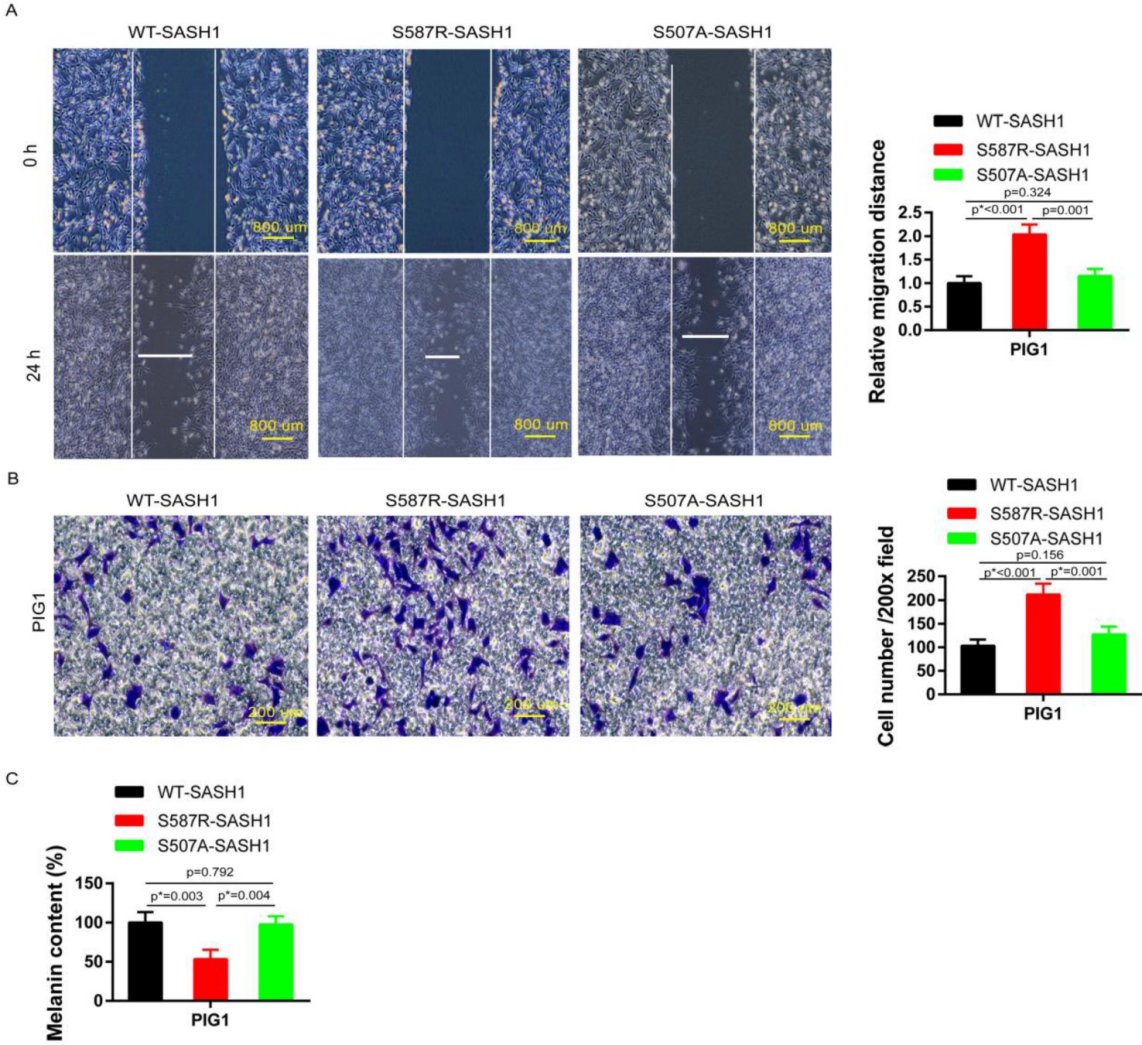
** S587R-SASH1 enhanced cellular migration and adhesion abilities, suppressed melanin biosynthesis in PIG1 cells. (A)** Wound-healing assays demonstrated 2.04 fold and 1.77 fold increase in S587R-SASH1 transfected PIG1 cells, as compared to WT-SASH1 and S507A-SASH1 cells. **(B)** Transwell migration assay revealed 2.05 fold and 1.67 fold increase in the number of S587R-SASH1 PIG1 cells that migrated after a 24 h period, as compared to WT-SASH1 and S507A-SASH1 cells. **(C)** SASH1 mutants obviously reduced the melanin content to 53.1% and 54.6%, as compared to that in WT-SASH1 and S507A-SASH1.

Transwell and wound-healing assays were performed using S587R-SASH1, S507A-SASH1and WT-SASH1 PIG1 cells, respectively. The invasive S587R-SASH1 PIG1 cells were up-regulated by 2.05 fold and 1.67 fold, as compared with the WT-SASH1 and S507A-PIG1 cells *in vitro* (Figure 5B). The migration assay showed that the migration of the mutant S587R-PIG1 cells increased more significantly, as compared to the WT-SASH1 PIG1 cells by 2.04 fold, and S507A-PIG1 cells by 1.77 fold (Figure 5A).

To further explore the effects of SASH1 mutations on melanin synthesis of MCs, melanin content assays were performed. As compared to the WT-SASH1 and S507A-SASH1 PIG1 cells, the S587R-SASH1 mutant downregulated the melanin content to 53.1% and 54.6%, respectively (Figure 5C).

### SASH1 mutation reduces THBS1 expression and negatively regulates TGF-β1 signaling

**Figure 6:**
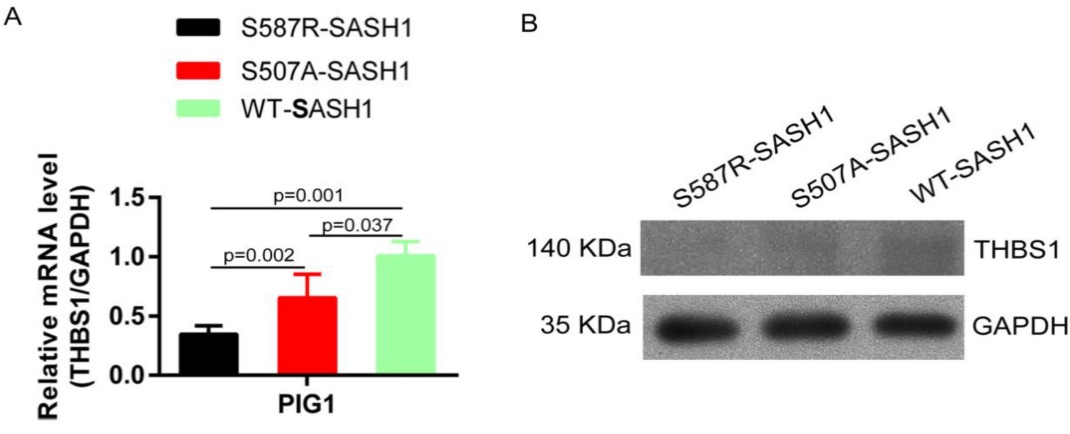
** SASH1 mutations down-regulated THBS1 expression. (A)** qPCR showed mRNA expression of THBS1 was reduced by 63.3% and 42.5% in S587R-SASH1 PIG1 cell, compared to the WT-SASH1 and S507A-SASH1 PIG1 cells. **(B)** Western blot revealed THBS1 protein was reduced by 74.2% and 48.5% in S587R-SASH1 PIG1 cells compared with WT-SASH1 and S507A-SASH1 PIG1 cells.

The endogenous expression of THBS1 mRNA in S587R-SASH1 PIG1 cells was reduced by 63.3% and 42.5%, respectively, as compared with the WT-SASH1 and S507A-SASH1 PIG1 cells. Western blot analysis further validated that THBS1 was reduced by 74.2% and 48.5% in mutant S587R-SASH1 PIG1 cells compared with WT-SASH1 and S507A-SASH1 PIG1 cells (Figure 6). We further investigated the response of SASH1 to TGF-β1 was investigated *in vitro*. Briefly, PIG1 cells were treated with 100 pmol/L TGF-β1 for 24 h and the expression of SASH1 was detected by western blot analysis. As shown in Figure 4B, the protein expression of SASH1 was decreased by 52.1% in response to TGF-β1.

## Discussion

Previous studies have reported that the ABCB6 gene in the 2q33.3-q36.1 region is responsible for DUH 6. However, DUH is a heterogeneous disease and a large portion of patients do not carry the ABCB6 variant 18. In the current study, exome sequencing with linkage mapping identified the SASH1 S587R variant as a plausible cause of DUH. The SASH1 gene is located at 6q24.3 and is 279,746 base pairs in length, which includes 22 exons and 21 introns, and encodes scaffold proteins of 1230 amino acids that contain two nuclear localization signals, a SLY domain, a SH3 domain, and two SAM domains 19-20. To date, the most commonly seen hotspot mutation region is located in the highly conserved SLY domain (382-536) 16. We choose the heterozygous c.1519T>G (p.Ser507Ala) reported to cause pigmentary abnormalities in SASH1 SLY domain as a control to determine MC function 14. The S587R-/S507A-SASH1 mutation was found to down-regulate the protein expression of SASH1. These two SASH1 mutants enhanced both the invasion and migration capacities, as well as suppressed melanin synthesis in PIG1 cells. Compared to the SASH1 SLY domain mutant, S587R-SASH1 exhibited reduced SASH1 expression, increased migration and invasion capacity and a less melanin content. SASH1 may promote cell migration through the TGF-β1 pathway, thereby contributing to abnormal MC morphology and localization in the epidermis.

We detected SASH1 expression throughout the skin tissues, including keratinocytes, fibroblasts, and especially MCs. Cellular experiments showed that SASH1 localizes to the nucleus and cytoplasm of epithelial cells. Although the central SH3 domain of the SASH1 protein might interact with the actin cytoskeleton and cortactin, a major regulator of actin filament dynamics in the regulation of cell migration and adhesion 21, the cell migration assay showed that fibroblasts with SASH1 variants had greater active migratory behavior than the control cells 17. Martini et al. showed that SASH1 plays an important role in tumor formation by regulating the migration and adhesion of cancer cells 21. Chen et al. used a transwell invasion chamber to investigate the effects of the SASH1 gene on A549 cell migration and found that the SASH1 gene may have an inhibitory effect 22. In that study, downregulation of the SASH1 gene led to a higher capability of migration and invasion of A549 cells and this negative regulation may be due to the involvement of the SASH1 gene in molecular pathways controlling cell migration and invasion. Zhou et al. showed that SASH1 variants may disrupt MC-keratinocyte adhesion by reducing E-cadherin expression, which leads to increased motility and altered melanin transfer of A375 cells 13.

In this study, the S587R substitution was located in the highly conserved SH3 domain, which was first described in the non-receptor tyrosine kinase Src involved in tyrosine kinase signaling and enzyme complexes 23-24. THBS1 is recognized as an extracellular homotrimeric protein that mediates antiangiogenic effects in endothelial cells by triggering the TGF-β1signaling cascade via the cell surface receptors CD36 and CD47 25. The N terminal KRFK sequence of THBS1 combined with the LSKL sequence in the N terminal 54-57 nucleotides of the latency-associated protein (LAP) form the THBS1/LAP-TGF-β1 complex, which specifically activates the TGF-β1 pathway 26. Wang confirmed the inhibitory effect of the activation of L-TGF-β1, which blocks the migration of THBS1-induced vascular smooth muscle cells 27. The CSVTCG sequence in the functional fragments thrombospondin type I repeats (TSR) TSR2 and TSR3 of THBS1 have stronger affinity to attach to the CD36 cell surface receptor. The combination of the CD36/THBS1/L-TGF-β1 complex with the cell surface receptor is reportedly essential for the release of TGF-β1 from the LAP and its biological activation 28. Several studies have revealed that the carboxyl terminal domain of CD36 forms a complex with Src family kinases, which is involved in signal transduction mediating cell adhesion, migration, and invasion in both human and mouse cell lines 29-30. The addition of phorbol 12-myristate 13-acetate downregulated TSR-mediated Src recruitment to CD36 on the surface of melanoma cells, suggesting that CD36 is required for TSR-mediated src recruitment to THBS1/L-TGF-β1 signaling pathway 28,29. Thus, we propose that the SASH1 S587R mutant in the SH3 domain causes abnormal binding to CD36, which is also a receptor for THBS1, which affects the conformational stability the of SH3/THBS1/L-TGF-β1 complex in the regulation of MC migration by promoting TGF-β1 activation and the release from LAP^31^,^32^.

Here, an investigation of a Chinese family with DUH showed that SASH1 was the causative mutation, which resulted in the significant down-regulation of protein expression. We also report for the first time the association of SASH1 with the migration of PIG1 cells via regulation of TGF-β1 signaling *in vitro*. These results highlight a new mechanism in some pigmentary skin diseases. Furthermore, our preliminary data results also demonstrated a correlation between SASH1 and THBS1 in PIG1 cells. THBS1 potentially acts as the key negative regulator of CD36/SH3/THBS1/TGF-β1 cascade. The ongoing follow-up studies mainly focus on the regulation mechanism in cell migration.

## Supplementary Material

Supplementary figures and tables.

## Abbreviations

DUH: Dyschromatosis universalis hereditaria
SASH1: SAM and SH3 domain containing 1
MCs: melanocytes
THBS1: thrombospondin 1
ABCB6: ATP binding cassette subfamily B member 6
TGF-β1: transforming growth factor beta 1, SNVs, Single nucleotide variants
qPCR: quantitative PCR
CADD: Combined Annotation Dependent Depletion
PolyPhen: Polymorphism Phenotyping
GATK: Genome Analysis ToolKit
LAP: latency-associated protein
TSR: thrombospondin type I repeats
HapMap: haplotype map
UCSC: University of California Santa Cruz
