# Supplementary material for: SASH1 promotes melanin synthesis and migration via suppression of TGF-β1 secretion in melanocytes resulting in pathologic hyperpigmentation
Source: Int J Biol Sci. 2020 Feb 10;16(7):1264–73. doi: 10.7150/ijbs.38415 (PMC7053321; doi:10.7150/ijbs.38415)
Supplement: Supplementary file 1 — Supplementary figures and tables. [file ijbsv16p1264s1.pdf]

Supplementary Materials

Supplementary Figure 1. The pathological examination of skin biopsy in the proband. A and B revealed the presence of dense pigment granules in hyperpigmented area and few or no pigment granules in the basal layer in hyper- and hypo-pigmented area, respectively. A: HE Stain, 100× magnification, Scale bar: 5 μ m; B: HE Stain, 200× magnification, Scale bar: 10 μ m.

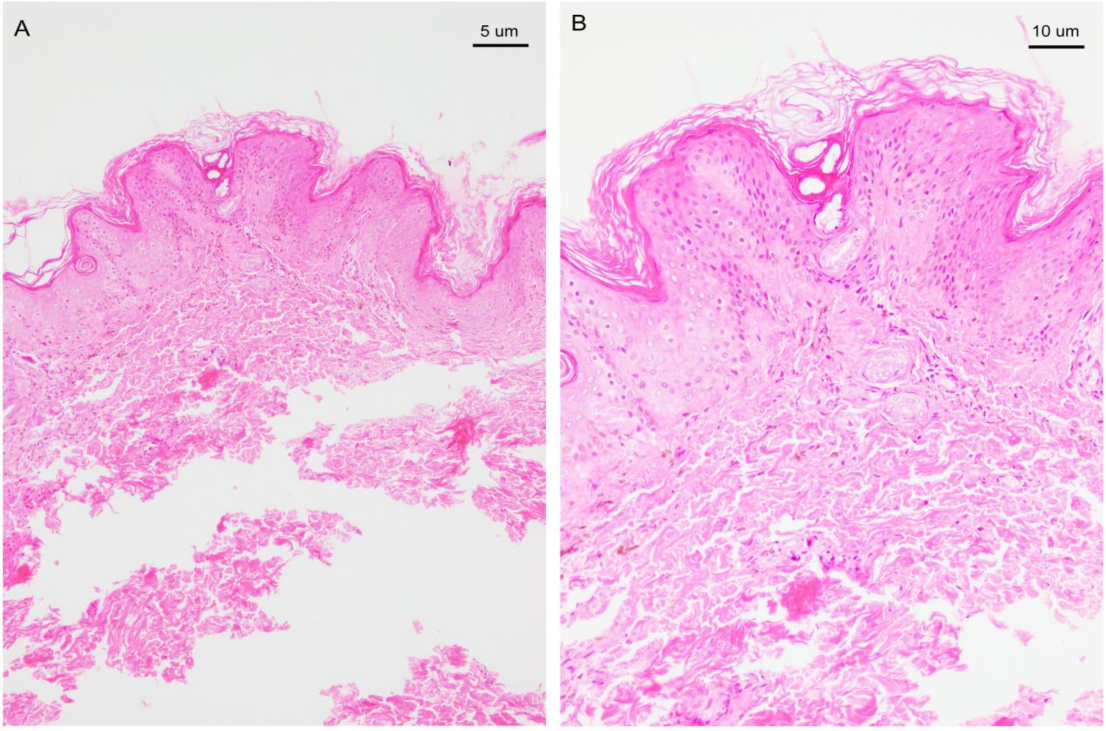

Supplementary Figure 2.The canonical data set of TGF-β1 and predicted downstream molecules. The orange and blue lines indicate the activating and inhibitory expression status between TGF-β1 and the downstream genes.

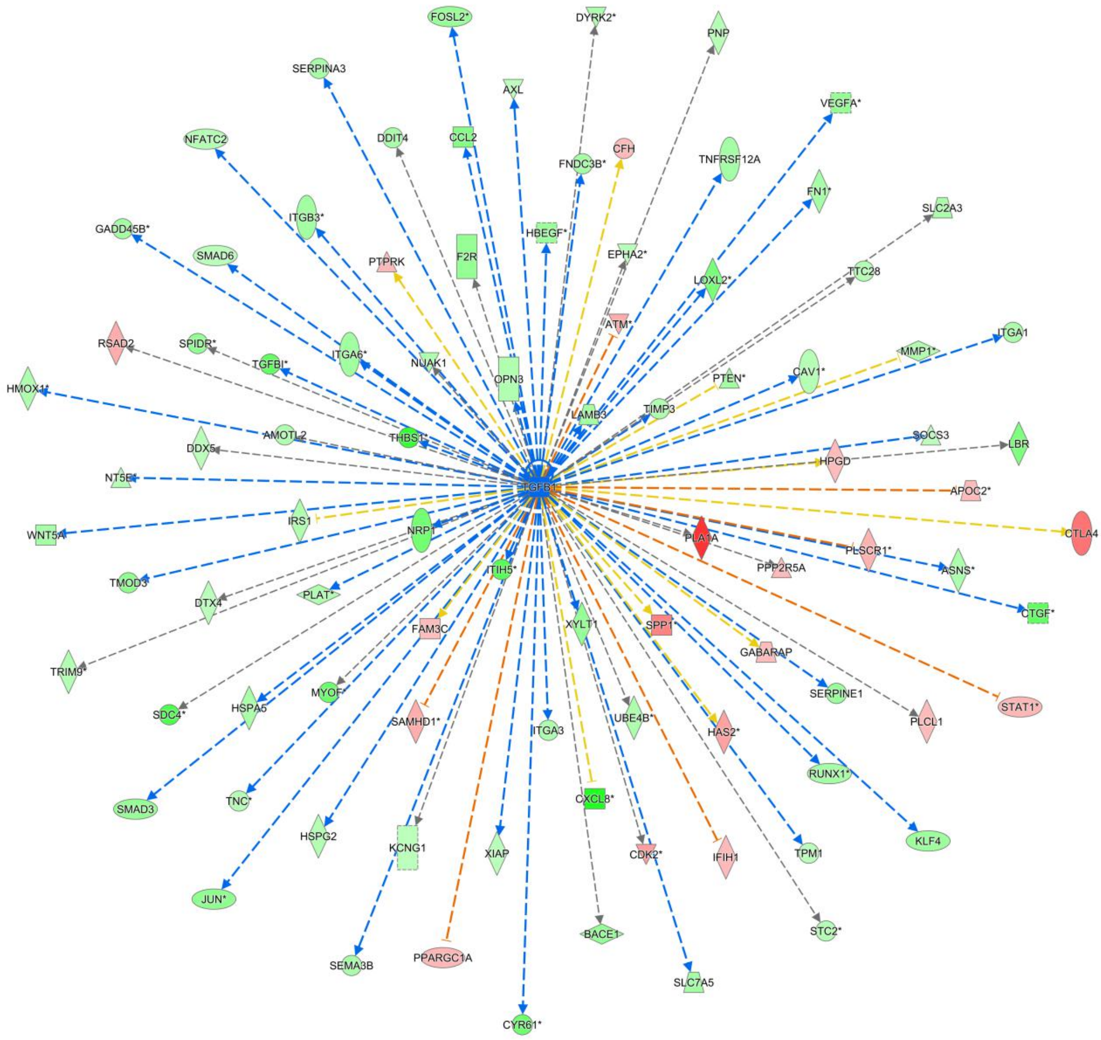

Supplementary Figure 3. Regulator effect network containing the paths from regulator to TGF-β1. The network showed a high consistency score, indicating the more accurate the result of regulation between TGF-β1 and cell migration.

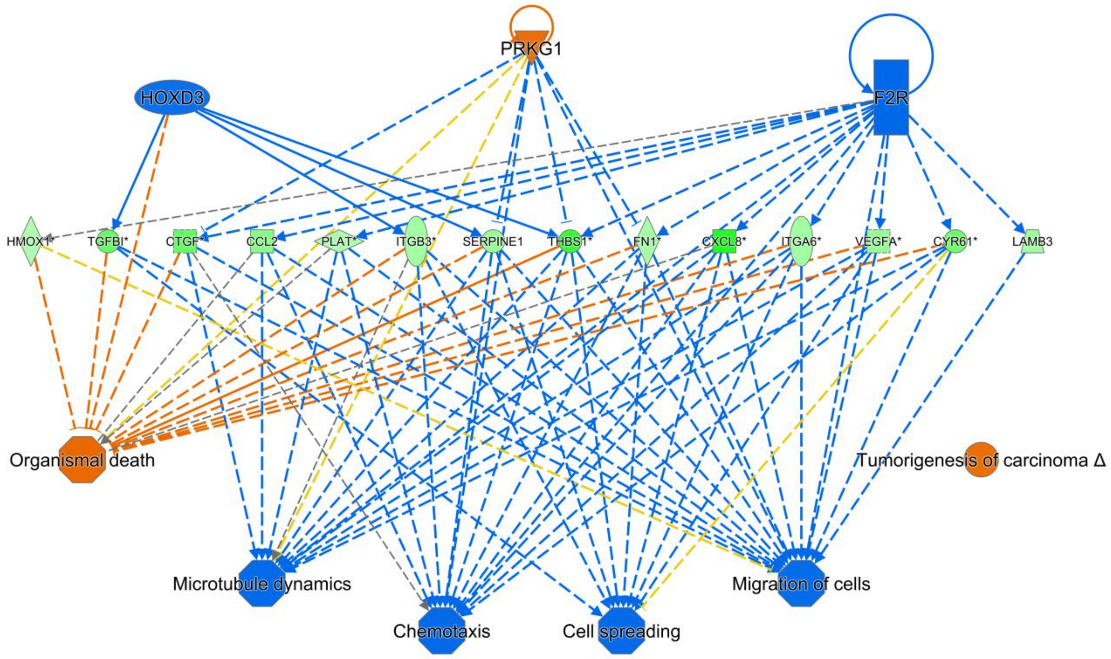

Supplementary Table 1. Summary of two exome sequencing data production.

Sample:	DUH-III:10	DUH-II:2
Total clean reads	103072538 (100%)	78887144 (100%)
Reads mapped to genome	102950330 (99.88%)	78774829 (99.86%)
Data mapped to genome(Mb)	12617.27	10049.09
Data mapped to target region (Mb)[a]	7556.27	6366.52
Fraction_of_effective_bases_on_target	59.9%	63.4%
Fraction_of_effective_bases_on_or_near_target	77.1%	79.0%
Average_sequencing_depth_on_target	124.99	105.31
Bases_covered_on_target	60391687	60387493
Coverage_of_target_region	99.9%	99.9%
Fraction_of_target_covered_with_at_least_100x	48.6%	39.1%
Fraction_of_target_covered_with_at_least_50x	82.2%	70.9%
Fraction_of_target_covered_with_at_least_20x	98.2%	95.8%
Fraction_of_target_covered_with_at_least_10x	99.6%	99.1%
Fraction_of_target_covered_with_at_least_4x	99.8%	99.8%

[a] Target regions here refer to the regions that are actually covered by the designed probes.

Supplementary Table 2. The screening process in identifying the potential mutations from exome data.

	DUH-III:10	DUH-II:2
Total SNPs & Indels	267113 & 35969	191063 & 22654
Potential SNPs & Indels[a]	23248 & 710	23360 & 680
Filtered_dbSNP_1000genomes_Deleterious[b]	1115 & 611	
Filtered_dbSNP_1000genomes_Deleterious_Model [c]	324SNVs in 280 genes &139 Indels in 114 genes)	
Within the linkage region[d]	five genes(*CPS1, SASH1, PTPRQ, CEP290, C12orf42*)	
Mutation analysis	c.1761C>G (p.Ser587Arg) in exon 15 of *SASH1*	

[a] Potential SNP & Indels includes nonsynonymous variants, splicing site variants and insertion/deletion variants located in exonic region.
[b]Variants were firstly excluded if they were present in the dbSNP (v.138 and v.142) database, the 1000 Genomes Project, the National Heart, Lung, and Blood Institute Exome Sequencing Project (ESP) Exome Variant Server (EVS), the Exome Aggregation Consortium (ExAC) Browser or the Novogene human WES & WGS databses**.**The filtered variants were evaluated with in silico tools SIFT, PolyPhen-2, MutationTaster and CADD, the candidates were predicted deleterious in at least 2 of the tools.
[c]On the basis of the autosomal-dominant genetic model in family pedigrees, we filtered compound heterozygous variants occurred in all affected members, but not in any of the unaffected individuals of the family.
[d] The linkage regions refer to chromosome 6q24.2-q25.2, 12q21-23 and 2q33.3-36.1 reported previously.

Supplementary Table 3. The screening process in identifying the potential mutations from exome data.

Variants	chromosomal locus	Gene	dbSNP ID	AF in 1000genomes	AF in NHLBI-ESP	AF in ExAC	AF in Novogene WES	AF in Novogene WGS	SIFT	Polyphen2	MutationTaster	CADD
chr2: 211521242CT>T	2q34	CPS1					0.0099					
chr6:148854933C>G	6q24.3	SASH1	rs147541734			0.0004			0.0020,D[1]	0.9760,D[2]	1.0000,D[3]	6.2385,28.8
chr12: 80899857T>C	12q21.31	PTPRQ	rs200063017	0.0032	0.0000	0.0004	0.0057	0.0276	0.0080,D[1]	0.8030,P[2]	0.9871,D[3]	5.0900,25.3
chr12: 88524335C>T	12q21.32	CEP290	rs10778257		0.5798	0.6458			0.0120,D[1]	0.0110,B[2]	0.6943,N[3]	4.2669,23.9
chr12: 103872172T>G	12q23.3	C12orf42					0.3324	0.3565	0.0000,D[1]	0.0720,B[2]	1.0000,P[3]	2.5391,19.7

Abbreviations: AF, allele frequency; NHLBI-ESP, National Heart, Lung and Blood Institute-Exome sequencing program; ExAC, Exome Aggregation Consortium; WES, whole exome sequencing; WGS, whole genome sequencing; D[1], Deleterious (sift<=0.05); D[2], Probably damaging (>=0.909); P[2], Possibly damaging (0.447<=pp2_hvar<=0.909); B[2], Benign(pp2_hvar<=0.446); D[3], Disease_causing; N[3], Polymorphism; P[3], Polymorphism_automatic.

Supplementary Table 4. The sequencing primers designed for the five potential mutations from exome data.

Primer Name	Strand	Sequence (5’-3’)	Product Size
CPS1: exon31:c.3577-6G>-	F	GCACAGAGAGGCAGCATTTT	767bp
	R	GTCCACACCTGTTTCACCAC	
SASH1: exon15:c. 1761 C >G	F	AGGTCACTCAGAGGGGTGA	580bp
	R	CTTACGCTATCACCCACAGC	
PTPRQ: exon12:c.T1811C	F	TCTGTGTGTTCTTCAGTGCA	919bp
	R	GAGCACTCCGAACTCCTTCA	
CEP290: exon8:c.G503A	F	CATCTCAGGTCTATGCGGGT	647bp
	R	CGGTAGTCACTGTCTTCCCC	
C12orf42: exon2:c.A33C	F	CCAAAGCAAGCACCACCTAG	980 bp
	R	TAACCCAGTGAGCCCTGTTT	
